# A Follow-Up Investigation of Mental Health Among Discharged COVID-19 Patients in Wuhan, China

**DOI:** 10.3389/fpubh.2021.640352

**Published:** 2021-04-12

**Authors:** Li Li, Michael Shengtao Wu, Junxiu Tao, Weijun Wang, Jing He, Ru Liu, Juan Guo, Yun Chen, Kejian Li, Shilong Li, Bo Qi, Buxin Han

**Affiliations:** ^1^Hubei Provincial Hospital of Traditional Chinese Medicine, Wuhan, China; ^2^School of Sociology and Anthropology, Xiamen University, Xiamen, China; ^3^Zhumadian Psychiatric Hospital, Zhumadian, China; ^4^Key Laboratory of Mental Health, Institute of Psychology, Chinese Academy of Sciences, Beijing, China

**Keywords:** COVID-19, quarantine, insomnia, anxiety, depression

## Abstract

**Objective:** To understand the mental health status and its risk factors among discharged COVID-19 patients during the first month of centralized quarantine and the subsequent home isolation.

**Methods:** The scales of the Insomnia Severity Index (ISI), General Anxiety Disorder (GAD-7), and Patient Health Questionnaire (PHQ-9) were used to measure the symptoms of insomnia, anxiety, and depression in 782 COVID-19 patients during the first month of centralized quarantine (March 16 to 26, 2020) and then during home isolation (April 3 to 10, 2020).

**Results:** During the centralized quarantine, the prevalence rates of insomnia, anxiety, and depressive symptoms were 44.37, 31.59, and 27.62%, respectively, and those during the home isolation decreased significantly at 27.11, 17.26, and 16.11%, respectively. In both waves, women showed a higher prevalence of symptoms of poor mental health compared to men, and middle-aged (40–59 years old) and elderly (≥60 years old) showed a higher risk of symptoms of poor mental health compared to the younger. In addition, the severity of COVID-19 revealed no significant relationship to symptoms of poor mental health, whereas, the interaction analysis revealed that those with other underlying diseases showed more symptoms of poor mental health during the centralized quarantine and a greater decrease during the follow-up home isolation.

**Conclusion:** The discharged COVID-19 patients suffered from mental health problems such as, insomnia, depression, and anxiety, and this was especially so for women, the middle-aged and elderly, and those with underlying diseases, but along with the rehabilitation and the environmental change from centralized quarantine to home isolation, all the mental symptoms were significantly alleviated. Based on a follow-up investigation, the current results provide critical evidence for mental health and early rehabilitation upon the discharged COVID-19 patients.

## Introduction

Major epidemics have a serious impact on both physical and mental health. Since December 2019, cases of coronavirus disease 2019 (COVID-19) have been detected in Wuhan, other regions of China, and many overseas societies. Exposed to the critical infectious disease (1), the general public and COVID-19 patients suffered from the spread of the epidemic and the psychological stress caused by the major social crises, such as, virus threats, drug interventions, life changes, and isolation restrictions.

However, the infectiousness of COVID-19 makes it difficult for researchers to reach patients directly and continuously. Most epidemic-related psychological studies are mainly concerned with ordinary residents and medical staff, and previous psychological studies on COVID-19 patients have just conducted a single, cross-sectional survey during their hospitalization, while there are very few follow-up investigations of mental health among discharged patients. In addition, it remains unclear what risk factors exist for the mental health of COVID-19 patients. Whether the gender, age difference, and disease exposure model in the classic theory can be validated in this group is debatable. For example, men and the elderly have no advantage in the face of infectious diseases, such as COVID-19, unlike ordinary diseases or life stress events. They are more susceptible to viruses, and viral infections and some experimental drug treatments will aggravate the underlying diseases and physical and mental pain (2).

More importantly, there is a lack of systematic evidence and longitudinal follow-up on how the discharged COVID-19 patients' mental health manifests along with the disease course and the environmental changes of quarantine (3). Therefore, a follow-up investigation of the mental health status and related risk factors of discharged COVID-19 patients in Wuhan are conducted during the first month of centralized quarantine and subsequent home isolation so as to provide a scientific basis and practical reference for the physical and mental rehabilitation of COVID-19 patients and psychological counseling under the epidemic situation.

## Methods

This research protocol was approved by the Ethics Committee of Hubei Hospital of Traditional Chinese Medicine (No. HBZY2020-C01-01), and the informed consent was obtained from the patients during the centralized quarantine (first wave) and then the home isolation (second wave).

### Subjects

The G^*^Power 3.1 was performed to run a power analysis, which indicated that a sample size of 330 is required to obtain an average power of 0.75 to detect an effect size of 0.03 (ρ2) at the standard 0.05 alpha error probability for a two-tail linear multiple regression analysis with three tested predictors (e.g., quarantine status, medical history, and their interaction) and seven predictors totally (including the other four, such as gender, age, cigarette smoking, and alcohol drinking)^1^.

A total of 1,148 discharged COVID-19 patients in Wuhan were recruited at three quarantine stations during the centralized quarantine from March 16 to 26, 2020, and during subsequent home isolation from April 3 to 10, 2020, but 366 (31.88%) failed to complete the questionnaire or did not participate the follow-up survey, resulting in 782 (68.12%) valid cases, aged 18 to 74 years old. For more details about demographic information (e.g., gender and age) and the level of COVID-19 severity (e.g., mild, moderate, and severe or critical), see Table 1.

**Table 1:** Demographic, medical and behavioral information of the discharged COVID-19 patients.

**Variables**		***n***	***%***
Gender	Male	402	51.4
	Female	380	48.6
Age	18~39	185	23.7
	40~59	372	47.6
	≥60	225	28.8
Severity^a^	Mild	340	43.5
	Moderate	273	34.9
	Severe or critical	79	10.1
	Missing	90	11.5
Medical history	Yes	329	43.1
	No	434	56.9
Cigarette smoking	Yes	114	14.9
	No	649	85.1
Alcohol drinking	Yes	146	19.1
	No	617	80.9

a *Severity indicates the clinical classification of COVID-19 (1). The mild cases show mild clinical symptoms, and there was no sign of pneumonia on imaging. The moderate cases show fever and respiratory symptoms with radiological findings of pneumonia. The severe cases meet any of the following criteria: (1) respiratory distress (≧30 breaths/min); (2) oxygen saturation ≤ 93% at rest; (3) arterial partial pressure of oxygen (PaO2)/fraction of inspired oxygen (FiO2) ≦300 mmHg (l mmHg = 0.133 kPa). The critical cases meet any of the following criteria: (1) respiratory failure and requiring mechanical ventilation; (2) shock; (3) any other organ failure that requires ICU care*.

### Materials and Procedures

The questionnaire included self-reported scales, which were used to measure the mental health symptoms (e.g., insomnia, anxiety, and depression) among COVID-19 patients. Furthermore, participants reported their previous medical history, covering hypertension, cerebral infarction, heart disease, emphysema, bronchial asthma, viral hepatitis, thyroid disease, diabetes, gout, fatty liver, gallstones, kidney disease, etc., In addition, The patients' behavioral (e.g., cigarette smoking and alcohol drinking) information was also reported, as shown in Table 1. On average, participants took about 15 min to complete the questionnaire.

For the 7-item Insomnia Severity Index (ISI), subjects were asked to report their insomnia symptoms over the past 2 weeks (e.g., “difficulty falling asleep or difficulty staying asleep”) using a 0 to 4 (0 = no and 3 = severe) Likert scale. The total score of the scale was from 0 to 28, with a score of 8 or higher indicating probable symptoms of insomnia (4). In the current study, the reliability (Cronbach's α) was 0.94 in the first wave and 0.95 in the second wave.

The 7 items on the subject of generalized anxiety disorder (GAD-7) (e.g., “upset, worried, and irritable”) were used to measure anxiety symptoms over the past 2 weeks. Subjects responded from 0 to 3 (0 = not at all and 3 = almost every day), with a score of 5 or higher indicating probable symptoms of anxiety (5–7). The reliability coefficients (Cronbach's α) were 0.95 in the first wave and 0.96 in the second wave, respectively.

The 9 items of the Patient Health Questionnaire (PHQ-9) (e.g., “I can't work hard or have no interest”) were used to measure depressive symptoms over the past 2 weeks, and subjects responded with a score from 0 to 3 (0 = not at all and 3 = almost every day), with a score of 5 or higher indicating possible depressive symptoms (5, 7, 8). The reliability coefficients (Cronbach's α) of the two waves were 0.87 and 0.95, respectively.

The above mental symptoms were measured twice, and all contact with patients was under the supervision of disease prevention and control headquarters in the quarantine stations. The interviewers, all the whom were registered psychiatrists, were trained to reach the participants face to face or by telephone, focusing on the following: (1) safe contacts during the questionnaire distribution; (2) relationship building with the vulnerable groups; (3) description about the questionnaire; (4) help with item understanding if needed; and (5) debrief and comfort skills. During the first wave, the screening was conducted during the centralized quarantine, about 14 ± 2 days after the discharge. Considering that some COVID-19's patients are elderly patients and did not have a smartphone, a paper-pencil questionnaire and face-to-face interview were employed. During the second wave, the screening was conducted during the home isolation, which was about 28 ± 2 days after discharge. Considering the highly possible risk of inflection during a home visit, a telephone-based interview instead of a face-to-face interview was employed.

Those who failed to complete the clinical scales or the follow-up survey were not included in the analysis, whereas the participants whose information about disease severity was missed were called again to double check. There were 90 patients who did not know the information of their disease severity who were included in the analysis of the difference in mental symptoms between the two waves but not in the regression models. The patients' privacy was restrictively protected, for which all the analyses were conducted anonymously and personal information (e.g., name, address, and cellphone number) was deleted before the data analysis.

## Results

### The Prevalence Rate of Poor Mental Health Symptoms During the Centralized Quarantine and Home Isolation

**Figure 1:**
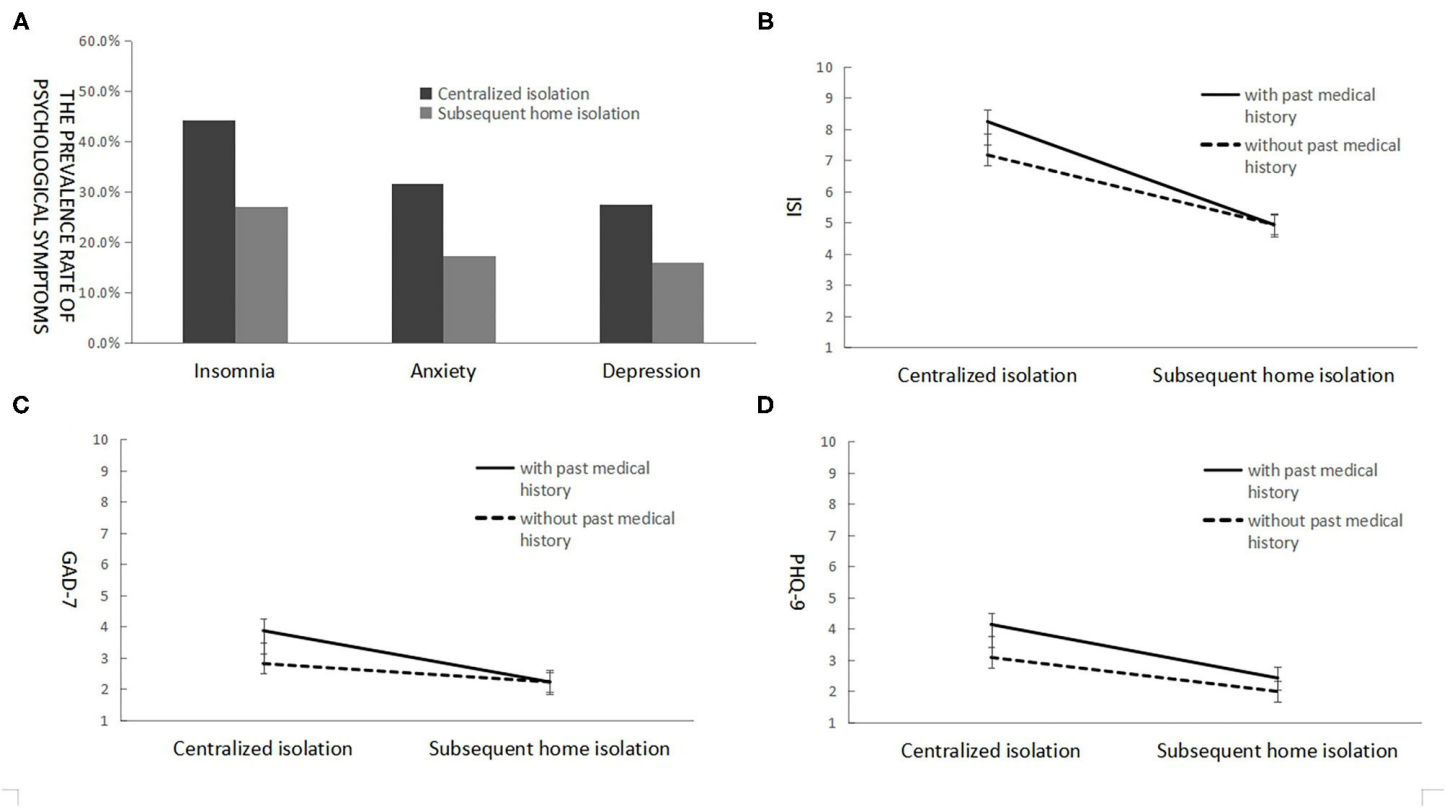
The prevalence rate of mental symptoms among the discharged COVID-19 patients during the first month of centralized quarantine and home isolation **(A)**, with the scores of insomnia **(B)**, anxiety **(C)**, and depression **(D)** depending on quarantine status and previous medical history.

As shown in Table 2, the prevalence rates of insomnia, anxiety, and depressive symptoms among discharged COVID-19 patients during the centralized quarantine period were 44.37, 31.59, and 27.62%, respectively, and the prevalence rates during the home isolation were 27.11, 17.26, and 16.11%, respectively. As shown in Figure 1A, paired-sample McNemar tests showed that the prevalence rate of mental symptoms during the home isolation significantly decreased, as compared with those during the centralized quarantine (*p* < 0.001). In addition, the prevalence rate of comorbidity (having insomnia, anxiety, and depressive symptoms at the same time) was 14.5% in the first wave, but this significantly decreased to 9.0% in the second wave (*p* < 0.001).

**Table 2:** Prevalence (%) of insomnia, anxiety, and depressive symptoms among the discharged COVID-19 patients across gender and age groups.

	**Insomnia1**^**a**^	**Insomnia2**^**a**^	**Anxiety1**^**b**^	**Anxiety2**^**b**^	**Depression1**^**c**^	**Depression2**^**c**^
**Gender**						
Male	148 (18.93)	84 (10.74)	84 (10.74)	58 (7.42)	85 (10.87)	43 (5.50)
Female	199 (25.45)	128 (16.37)	163 (20.84)	77 (9.85)	131 (16.75)	83 (10.61)
**Age**						
18~39	48 (6.14)	29 (3.71)	34 (4.35)	21 (2.69)	32 (4.09)	17 (2.17)
40~59	177 (22.63)	105 (13.43)	126 (16.11)	73 (9.34)	114 (14.58)	62 (7.93)
60~79	122 (15.60)	78 (9.97)	87 (11.13)	41 (5.24)	70 (8.95)	47 (6.01)
Total	347 (44.37)	212 (27.11)	247 (31.59)	135 (17.26)	216 (27.62)	126 (16.11)

a *ISI scores 8 or above*;

b *GAD-7 scores 5 or above*;

c *PHQ-9 scores 5 or above*.

Though the discharged patients' mental symptoms significantly decreased, there were some who failed to recover when moving from the centralized quarantine to the home isolation. Although, 159 patients (20.3%) with insomnia symptoms, 169 patients (21.6%) with anxiety symptoms, and 132 patients (16.9%) with depressive symptoms recovered along with the rehabilitation and the environmental change, there were 188 patients (24.0%) with insomnia symptoms, 78 patients (10.0%) with anxiety symptoms, and 84 patients (10.7%) with depressive symptoms who suffered from mental health problems in both two waves.

### Correlates of Mental Symptoms

Pearson correlation analysis showed that gender, coded as 1 (woman) and 0 (man), was significantly positively correlated with mental symptoms, among which the scores of anxiety were relatively higher among women than men, both during the centralized quarantine and home isolation. There was a significant positive correlation between age and mental symptoms, and the scores of insomnia, anxiety, and depression were relatively higher in the elderly than in the younger. However, there was no significant correlation between disease severity and mental symptoms, whereas previous medical history (1 = yes and 0 = no) was significantly positively correlated with all the three mental health symptom scores of insomnia and anxiety during the centralized quarantine but with depressive symptoms only during the home isolation.

Surprisingly, smoking and drinking were negatively related to symptoms of poor mental health, with smokers and drinkers having fewer symptoms. In addition, the poor mental health symptoms were also significantly and positively correlated with each other (see Table 3).

**Table 3:** Correlation analysis among variables.

**Variable**	**2**	**3**	**4**	**5**	**6**	**7**	**8**	**9**	**10**	**11**	**12**
1 Gender^a^	0.06	0.01	0.01	−0.36^**^	−0.41^**^	0.19^**^	0.16^**^	0.23^**^	0.14^**^	0.17^**^	0.19^**^
2 Age	1	0.13^**^	0.37^**^	−0.06	−0.02	0.27^**^	0.20^**^	0.17^**^	0.08^*^	0.15^**^	0.12^**^
3 Severity^b^		1	0.07	−0.08^*^	−0.05	0.04	0.04	0.01	−0.01	0.03	0.02
4 Medical History^c^			1	−0.00	−0.04	0.16^**^	0.07	0.16^**^	0.03	0.15^**^	0.09^**^
5 Smoking^c^				1	0.45^**^	−0.14^**^	−0.11^**^	−0.15^**^	−0.08^*^	−0.12^**^	−0.11^**^
6 Drinking^c^					1	−0.11^**^	−0.08^*^	−0.11^**^	−0.09^*^	−0.09^*^	−0.09^*^
7 Insomnia1						1	0.62^**^	0.59^**^	0.34^**^	0.67^**^	0.48^**^
8 Insomnia2							1	0.41^**^	0.61^**^	0.49^**^	0.76^**^
9 Anxiety1								1	0.40^**^	0.78^**^	0.49^**^
10 Anxiety2									1	0.38^**^	0.78^**^
11 Depression1										1	0.55^**^
12 Depression2											1

a *Gender is divided into 1 (women), 0 (men)*,

b *the clinical classification is 1 (light), 2 (normal), 3 (severe or critical)*,

c *and previous medical history, smoking, and drinking are unified as 1 (Yes), 0 (No)*;

** *p < 0.01*,

* *p < 0.05*.

### Effect of Medical History on the Symptom Changes

Using the symptom changes of insomnia, anxiety, and depression across the two waves as the dependent variable, the stepwise regression analyses were conducted, using the first step predictors (e.g., gender, age, smoking, drinking, COVID-19 severity, and medical history) and the second step predictors (e.g., the interaction between medical history with gender, age, and COVID-19 severity). The results showed that medical history significantly predicted the symptom changes of insomnia, anxiety, and depression, and gender significantly predicted the symptom changes of anxiety. However, we found no significant prediction effect of the above interaction variables, which all failed to be included in the regression model. Similarly, regarding the interaction between gender and smoking and the interaction between gender and drinking as the second step predictors, we still failed to find a significant prediction effect of the two interaction variables that could be included in the regression model.

For the significant effect of medical history, as shown in Figures 1B–D, the patients having baseline diseases reported more symptom changes of mental problems, as compared with those having no baseline disease. In addition, women also reported more symptom change of anxiety than men did (see Table 4).

**Table 4:** The prediction for symptom changes of insomnia, anxiety, and depression.

**Predictors**	**Insomnia**			**Anxiety**			**Depression**		
	**B**	**SE**	***t***	**B**	**SE**	***t***	**B**	**SE**	***t***
Gender^a^	0.31	0.49	0.63	0.78	0.38	2.05^*^	0.10	0.33	0.30
Age	0.03	0.02	1.77	0.01	0.02	0.86	0.01	0.01	0.66
Smoking^c^	−0.42	0.70	−0.59	−0.72	0.55	−1.33	−0.37	0.47	−0.78
Drinking^c^	−0.21	0.65	−0.32	0.42	0.50	0.84	−0.01	0.43	−0.03
Severity^b^	−0.13	0.32	−0.41	−0.03	0.25	−0.11	0.02	0.22	0.07
Medical History^c^	1.07	0.47	2.26^*^	1.06	0.37	2.89^**^	0.64	0.32	2.02^*^

a *Gender is divided into 1 (women), 0 (men)*;

b *the clinical classification is 1 (light), 2 (normal), 3 (severe or critical)*;

c *and previous medical history, smoking, and drinking are unified as 1 (Yes), 0 (No)*;

** *p < 0.01*,

* *p < 0.05*.

## Discussion

Follow-up investigations showed that more than 40% of discharged COVID-19 patients suffered from insomnia, and about 30% suffered from anxiety and depression during the centralized quarantine (in the first 2 weeks after discharge). The result was similar to the prevalence rate of insomnia and anxiety during hospitalization of the suspected or confirmed COVID-19 patients in the past literature, but the prevalence rate of depression among the discharged patients was lower than that (50%) among the hospitalized COVID-19 patients (9–11). Furthermore, the symptoms of poor mental health and their comorbidity during home isolation (about 1 month after discharge) decreased significantly in the discharged patients.

Comparing the prevalence rates of the three mental symptoms of patients, it was found that the prevalence rates of insomnia (44.37 and 27.11% in two waves) during the centralized quarantine period and the home isolation period were higher than the prevalence rates of anxiety (31.59 and 17.26%) and depressive symptoms (27.62 and 16.11%). This is possibly because the discharged patients suffered from breathing difficulties, medication interference, and experienced big changes in life rhythm during the centralized quarantine and subsequent home isolation, which damaged the patients' sleep quality, more so than emotional well-being. This suggests that insomnia after discharge from the hospital for COVID-19 patients should be more concerned during early treatment.

The majority of patients remained resilient, showing no obvious symptoms of insomnia, anxiety, or depression after discharge even though they had psychological stress, which may be related to the strong system of disease control and prevention and social support taken by the country after the outbreak of COVID-19. For example, all suspected and confirmed patients were admitted to the hospital and the fees of medical treatment and care were covered by the central government, and free food and accommodation were provided in the quarantine locations. Furthermore, mental symptoms were significantly decreased during the subsequent home isolation compared with the centralized quarantine, which suggested that being at home can improve the quality of sleep and emotional well-being, because protective factors matter, such as, familiar living environment and family support, which can effectively relieve stress and promote physical and mental health (12).

However, the overall decrease in the three mental health symptoms and their comorbidity along with the physical rehabilitation (and environmental change) does not mean that all the patients are free from the mental sufferings and do not need psychological treatment. The current findings showed that although 16.9–21.6% of patients recovered when moving from the centralized quarantine to the home isolation, there were still 10.0–24.0% of patients who suffered from chronic problems over time. This suggests that case-by-case observations and treatments should be seriously concerned in the follow-up screening and clinical practices upon chronic pains among the discharged COVID-19 patients.

Furthermore, this study also showed that patients who were women had higher prevalence rates of mental symptoms than men. In particular, in women, the prevalence rates of insomnia, anxiety, and depression were 1.34, 1.94, and 1.54 times those of men during the centralized quarantine, which was consistent with results of most previous studies on mental health (13). This might because women were relatively vulnerable in face of public health emergencies (12). Women's multiple social roles also matter. Smokers and drinkers had fewer mental symptoms, and this is possibly because most of the smokers and drinkers were men who had fewer symptoms of mental health problems than women did. Among the 782 patients in the current study, women over 40 years old accounted for 79% of the total number of females. Women in this age group had families and worried not only about their own health and jobs but also about the lives and health of the elderly, their husbands and children, and even about the online education of children or the support of grandchildren. In this vein, women should be given special support by psychological counseling and health promotion for COVID-19 patients, and their special role in family functions and epidemic prevention and control was also worthy of attention.

In addition, the prevalence rates of mental symptoms were significantly different across age groups, in that the symptoms were more serious in middle-aged and elderly people than in young people. Although middle-aged and elderly people had certain advantages in daily emotional regulation strategies (such as being better at maintaining interpersonal emotions and seeing the meaning of life), they were incapacitated in the face of major life crisis events and even more vulnerable to life shocks (14) and so felt greater psychological pressure (15). At the same time, the prevalence and mortality of COVID-19 in middle-aged and elderly people are relatively higher than the younger ones (2). The elder patients worried about their poor immunity and many underlying diseases and even about bringing burdens to their families, not being able to accompany their grandchildren to enjoy the happiness of family, or affecting their future life quality and being despair in later life.

It should be noted that the classification of the severity of the disease was not significantly correlated with mental symptoms during the centralized quarantine or the home isolation, and revealed no significant effect on the changes of mental symptoms in the two waves. These results did not support the previous model of disease exposure—the more severe the disease, the more symptoms there were (2, 3). However, the patients' previous basic medical history was significantly positively correlated with symptoms of insomnia, anxiety, and depression, especially during the centralized quarantine. This might because patients with basic diseases had poor immunity, more complications, and greater psychological pressure. In the handbook of COVID-19 diagnosis and treatment, clinicians also claimed that patients with chronic underlying diseases had a poor prognosis (1), which suggested that we should pay more attention to patients with underlying diseases in early psychological counseling and intervention upon the discharge of COVID-19 patients.

The limitations of this study include that the treatment plan, sequelae, and other medical background information as well as social and psychological factors, such as, marriage, occupation, education level, and family income, were not taken into account, which reduces the generalization of the current findings. In addition, several other factors weaken the strength and applicability of the current study: (1) no control group was considered, such as healthy controls, or patients who kept staying in centralized quarantine for a longer time when others were transferred to home isolation; (2) the efficacy or effect of psychological intervention (e.g., public mental health services) was not tracked, for which it remains unclear to what extent the decrease of mental symptoms was the results of the treatment or just the natural recovery along with physical rehabilitation; (3) given that mental problems are stigmatized in many cultures and the current study was mainly based on face-to-face or telephone interviews, the effect of social desirability matters but was not controlled.

Nonetheless, based on an early follow-up investigation among the discharged COVID-19 patients at the time when they were discharged in the centralized quarantine and the subsequent home isolation, the current works provide critical evidence for the impact of mental health and early rehabilitation upon discharged patients in the pandemic. We have found that the COVID-19 patients, especially women, the middle-aged, the elderly, and those with underlying diseases, suffered from insomnia, anxiety, and depressive problems after discharge. Therefore, in the process of disease prevention, control, and treatment, psychiatrists and clinical psychologists should play an active role in identifying the mental illness as soon as possible, early screening and care should be carried out for discharged patients, and special attention and care should be given more to vulnerable groups.

## Data Availability Statement

The raw data supporting the conclusions of this article will be made available by the authors, without undue reservation.

## Ethics Statement

The studies involving human participants were reviewed and approved by the Ethics Committee of Hubei Hospital of Traditional Chinese Medicine (No. HBZY2020-C01-01). The patients/participants provided their written informed consent to participate in this study.

## Author Contributions

LL: research design, data collection and analysis, paper writing, and revision. MW: research design, data analysis, paper writing, and revision. JT and WW: data collection and analysis. JH, RL, JG, YC, KL, SL, and BQ: data collection. BH: research design and paper revision. All authors contributed to the article and approved the submitted version.

## Conflict of Interest

The authors declare that the research was conducted in the absence of any commercial or financial relationships that could be construed as a potential conflict of interest.

## Footnotes

^1^If regarding all the seven predictors as the tested ones, the required sample size is 440, whereas the other four variables (e.g., gender, age, cigarette smoking, and alcohol drinking) were not our research focus.
